# 

**Published:** 1911-06

**Authors:** 


					﻿VOL. XXVI.
JUNE, 1911.
•Ko. 12^-
TEX AS
MEDIC/fc?
JOURNAL
“Red Back”
PUBLISHED AT AUSTIN, TEXAS. BY
F. E. DANIEL, M. D., - - - Editor and Proprietor.
PRINTED BY THE VON BOECKM ANN-JONES CO.
Subscription, Si.00 a Year, in Advance. -	-	-	- Single Copies, 10 Cents
Entered at the Postoffice at Austin, Texas as second class mail matter.
				

